# Effects of Different Spectral Shapes and Amplitude Modulation of Broadband Noise on Annoyance Reactions in a Controlled Listening Experiment

**DOI:** 10.3390/ijerph15051029

**Published:** 2018-05-19

**Authors:** Beat Schäffer, Reto Pieren, Sabine J. Schlittmeier, Mark Brink

**Affiliations:** 1 Empa, Laboratory for Acoustics/Noise Control, Swiss Federal Laboratories for Materials Science and Technology, 8600 Dübendorf, Switzerland; reto.pieren@empa.ch; 2 HSD Hochschule Döpfer—University of Applied Sciences, 50676 Köln, Germany; s.schlittmeier@hs-doepfer.de; 3 TU Kaiserslautern, 67663 Kaiserslautern, Germany; 4 Federal Office for the Environment, 3003 Bern, Switzerland; mark.brink@bafu.admin.ch

**Keywords:** annoyance, broadband sounds, spectral shape, wind turbine noise, low-frequency noise, amplitude modulation

## Abstract

Environmental noise from transportation or industrial infrastructure typically has a broad frequency range. Different sources may have disparate acoustical characteristics, which may in turn affect noise annoyance. However, knowledge of the relative contribution of the different acoustical characteristics of broadband noise to annoyance is still scarce. In this study, the subjectively perceived short-term (acute) annoyance reactions to different broadband sounds (namely, realistic outdoor wind turbine and artificial, generic sounds) at 40 dBA were investigated in a controlled laboratory listening experiment. Combined with the factorial design of the experiment, the sounds allowed for separation of the effects of three acoustical characteristics on annoyance, namely, spectral shape, depth of periodic amplitude modulation (AM), and occurrence (or absence) of random AM. Fifty-two participants rated their annoyance with the sounds. Annoyance increased with increasing energy content in the low-frequency range as well as with depth of periodic AM, and was higher in situations with random AM than without. Similar annoyance changes would be evoked by sound pressure level changes of up to 8 dB. The results suggest that besides standard sound pressure level metrics, other acoustical characteristics of (broadband) noise should also be considered in environmental impact assessments, e.g., in the context of wind turbine installations.

## 1. Introduction

Large portions of the population are exposed to hazardous (technical) environmental noise (e.g., [1,2]). While environmental noise is associated with various health impacts such as sleep disturbance or cardiovascular effects [2], noise annoyance is particularly widespread [3,4]. As the number of exposed people is likely to increase in the future, adequate environmental noise impact assessment becomes increasingly important.

Environmental noise from transportation or industrial infrastructure (e.g., wind farms) is typically broadband. Different sources may evoke annoyance reactions of different magnitudes [5,6], which is explainable by their differing acoustical characteristics. Such characteristics comprise (i) sound pressure level, (ii) spectral shape, and (iii) (very) short-term temporal level variations, referred to as amplitude modulation (AM).

Sound pressure level is crucial to annoyance (e.g., [5,6]) and, accordingly, the major variable in environmental noise impact assessment (e.g., [2,7]). Moreover, studies on the association of annoyance with spectral shape revealed that both low [8,9] and mid to high frequencies [10] may be important. However, studies comparing the effects of mid and high frequencies with those of low frequencies are scarce, and their findings are contradictory. One study found that annoyance increased with low-frequency content [11], while other studies stress the relative importance of high frequencies [12,13]. Furthermore, AM may be relevant. Modulation functions of AM may be quasi-periodic or random (or both). Periodic AM is sometimes observed for wind turbine (WT) noise and related to the blade passing frequency of WTs (e.g., [14]). It was found to be strongly associated with noise annoyance [15,16]. Also, random AM may play a role. For environmental noise, random AM can be caused by atmospheric turbulence, which affects sound emission [17] as well as propagation [18]. While omnipresent in the environment, we are not aware of any study investigating its effect on annoyance. Also, knowledge of the relative contribution of the above acoustical characteristics of broadband noise to annoyance is still scarce.

The objective of the present study was therefore to investigate short-term annoyance reactions to different situations of broadband sounds under controlled laboratory conditions (see also [19]). Parts of the situations consisted of realistic outdoor WT broadband sounds. WT was chosen as an environmental sound because, due to its strong effect on annoyance [5], it is a currently much discussed topic [20,21,22], at which several of our recent activities were also directed [23,24,25,26]. The WT sounds were complemented with artificial, generic broadband sounds. The study design allowed for separating the contributions of three acoustical characteristics to acute noise annoyance reactions, namely, spectral shape, depth of periodic AM, and occurrence (or absence) of random AM. While the outcomes generally apply to (environmental) broadband noise, practical implications may be specifically directed towards WT noise.

## 2. Materials and Methods

### 2.1. Listening Test Design

In this study, the effect of different acoustical characteristics of outdoor WT and other, generic broadband sounds on noise annoyance was studied under laboratory conditions. WT sounds were included because (i) WT noise effects are a much discussed topic, (ii) this study is a follow-up of a recent listening experiment by us, where annoyance with WT and road traffic noise was compared [26], and (iii) to put the annoyance assessment generally into environmental and specifically into WT noise context. The WT sounds were complemented with generic sounds instead of (further) realistic environmental sounds to have full control on the studied acoustical characteristics. The outcome corresponds to acute, “short-term” [27] or “psychoacoustic” [28] annoyance.

According to Swiss cantonal and federal law, this study was not subject to approval by an ethics committee. However, the Cantonal Ethics Committee KEK Zurich, after having checked the project, stated that from an ethical point of view there was no objection to carrying out the study (Waiver No. 40-2015 (KEK-ZH-No. 2014-0294) from 22 April 2015).

In the listening experiment, 18 acoustical stimuli were systematically varied with respect to three variables: (i) spectral shape; (ii) depth of periodic AM, expressed as standard deviation of the periodic level fluctuations (*σ*_pAM_); and (iii) occurrence or absence of random AM (Table 1).

The spectral shape covers a wide range from pink to a realistic WT to low-frequency (LF) spectral shape. Pink spectral shape (i.e., 1/f power distribution) was included as a broadband sound with the same energy in all 1/3 octave bands (flat spectrum in logarithmic frequency bands). It is well-defined, closer to environmental sounds than white noise and has been used as a reference sound in previous laboratory studies [11,27]. A typical WT spectrum was chosen as a broadband environmental sound with prominent low-frequency components (20 Hz to ~200 Hz) [29] and thus a distinct “spectral slope” of the (unweighted) sound pressure level with increasing octave band [30]. Finally, an “exaggerated” LF spectrum with a double WT spectral slope, i.e., strong low frequency components but weak mid and high frequency components, was included (cf. Section 2.2). Pink and LF are generic spectra. The depth of periodic AM covers the occurrence of no (*σ*_pAM_ = 0 dB), medium (*σ*_pAM_ = 1.5 dB) and strong AM (*σ*_pAM_ = 3 dB). For WT, the latter two represent situations with high-frequency “swishing” and mid-frequency “thumping” sound [14]. The occurrence or absence of random AM was studied to assess its contribution to annoyance compared to periodic AM. The stimuli were reproduced at a *L*_Aeq_ of 40 dBA, which is a typical WT noise exposure for residents living close to wind farms and already associated with annoyance reactions (e.g., [31,32]).

In addition, one WT sound was used as a reference and additionally reproduced at a *L*_Aeq_ of 37 and 43 dBA, besides 40 dBA (Table 1). This reference (three stimuli) was used to express the contribution of the above three variables to annoyance as equivalents of a (WT) sound pressure level change.

From the resulting set of 20 stimuli, two subsets were separately analyzed regarding annoyance (cf. Section 2.7), with the reference stimulus at a *L*_Aeq_ of 40 dBA (Table 1) included in both subsets:

- **Subset I** contained the ratings of the reference sound of Table 1 at a *L*_Aeq_ of 37, 40 and 43 dBA. It reveals the annoyance reactions to *L*_Aeq_ changes.
- **Subset II** contained the ratings of the 18 stimuli of Table 1 without the two additional stimuli at a *L*_Aeq_ of 37 and 43 dBA. It reveals the annoyance reactions to the variables of Table 1 at a constant *L*_Aeq_ of 40 dBA.

While the full factorial design of the experiment (Table 1) required the inclusion of situations that do not occur in reality (namely, situations with LF and pink spectral shape and/or without random AM), it allowed for separation of the effects of the three variables on annoyance.

### 2.2. Acoustical Stimuli

The acoustical stimuli of Table 1 were artificially generated using the sound synthesis technique and the data described in [24,25].

Emission synthesis of the WT stimuli represents one single 2 MW Vestas V90 (three blades, hub height = 95 m, rotor diameter = 90 m) at strong wind and without tonal components. The depth of periodic AM was modelled by adjusting *σ*_pAM_ to 0.0, 1.5 or 3.0 dB (Table 1). The fluctuation frequency was set to 0.75 Hz, corresponding to a rotational speed of 15 rpm for a WT with three blades. Random AM of the emission was either simulated with a frequency dependent standard deviation (*σ*_rAM_) amounting to ~1.5 dB at high frequencies [25] (stimuli “with random AM”) or switched off (“no random AM”). On the emission signals, propagation filtering [24] was performed for flat grassy terrain and a distance of 400 m, which corresponds to a *L*_Aeq_ of ~40 dBA. The receiver height was set to 2 m above ground. The filtering accounts for geometric spreading, air absorption and ground reflection from an extended source, as well as for random AM from propagation (turbulence in the case of WT) with a *σ*_rAM_ of 1.5 dB, but the latter only for the stimuli “with random AM” (otherwise switched off).

The stimuli with pink and LF spectral shapes were created by modifying the spectral shaping in the above described synthesis approach. Hereby, the LF spectrum was calculated based on the above resulting WT and the pink spectrum such as to obtain the same (absolute) sound level difference between LF and WT as between pink and WT in each 1/3 octave band.

The stimuli were normalized in amplitude to exactly match the desired *L*_Aeq_ of 40 dBA. In addition, the reference stimulus (cf. Table 1) was duplicated twice and scaled in amplitude to a *L*_Aeq_ of 37 and 43 dBA. For the experiments, a stimulus length of 20 s was chosen, which in [26] was found to be optimal. The 20 resulting synthesized sound pressure signals were saved as single-channel audio signals in the WAVE PCM format.

**Figure 1:**
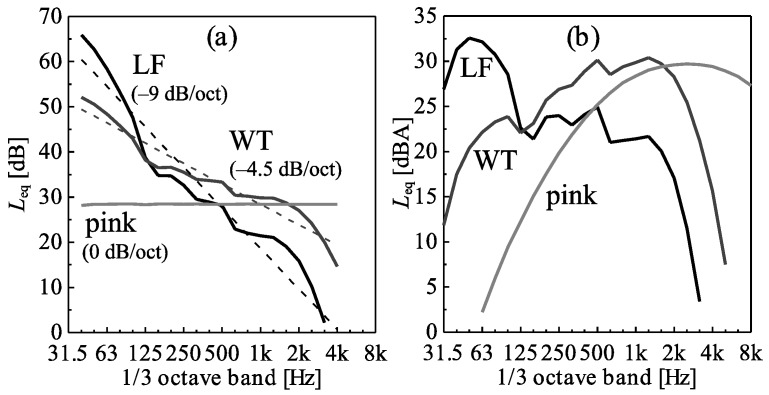
(**a**) Unweighted and (**b**) A-weighted 1/3 octave band spectra (in *L*_eq_, mean over the whole stimuli length) of the low-frequency (LF), wind turbine (WT) and pink spectral shapes for the situations without periodic and without random amplitude modulation, with a *L*_Aeq_ of 40 dBA. In (**a**), the mean spectral slopes (dashed lines: regressions of WT and LF *L*_eq_ on 1/3 octave band) are shown.

Figure 1 shows the resulting LF, WT and pink spectra for the situations without periodic AM and without random AM. The spectra of the other situations are identical. The WT spectrum is a typical one, lying within the bundle of WT spectra and having a spectral slope close to the −4 dB/oct measured in residential areas [30] (Figure 1a). LF has a double spectral slope of −9 dB/oct, and pink 0 dB/oct (Figure 1a). The differences *L*_C-A_ between the C-weighted sound pressure level (*L*_Ceq_) and *L*_Aeq_ of the stimuli amount to 2, 16 and 29 dB for pink, WT and LF spectra, respectively. This indicates that the WT and in particular the LF spectral shapes contain substantially more energy at low frequencies than the pink shape (Figure 1).

**Figure 2:**
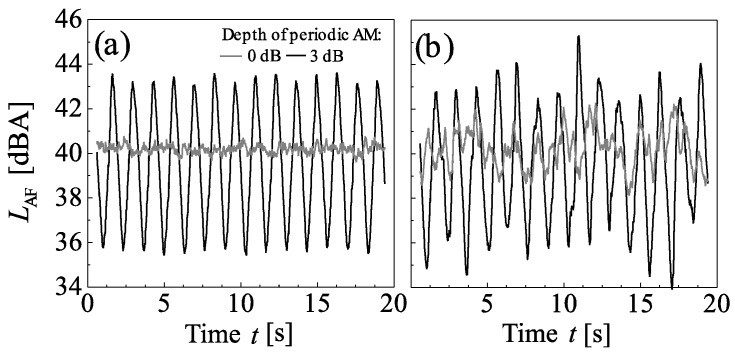
Level-time histories of the A-weighted, FAST time-weighted sound pressure level (*L*_AF_) of the stimuli with wind turbine spectral shape and a *L*_Aeq_ of 40 dBA, for depths of periodic amplitude modulation (AM) of 0 and 3 dB, in situations (**a**) without and (**b**) with random AM.

**Figure 3:**
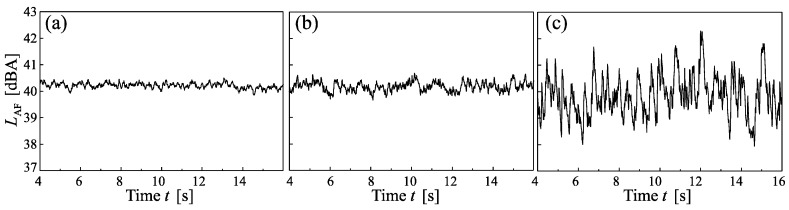
Level-time histories of the A-weighted, FAST time-weighted sound pressure level (*L*_AF_) of the stimuli without periodic and without random amplitude modulation, for (**a**) pink, (**b**) wind turbine and (**c**) low frequency spectral shapes.

Figure 2 and Figure 3 show exemplary level-time histories of the A-weighted, FAST time-weighted sound pressure level (*L*_AF_). Both periodic and random AM strongly affect the level-time histories of the stimuli. Periodic AM increases the standard deviation of the *L*_AF_ in situations with and without random AM (Figure 2), while random AM increases the standard deviation in situations without periodic AM (Figure 2b vs. Figure 2a). The effects of periodic and random AM are not additive, but in situations with periodic AM, random AM results in more irregular periodic level fluctuations than if no random AM is present (Figure 2b vs. Figure 2a).

Besides, the level-time histories also depend on spectral shape (Figure 3), with the standard deviation of the *L*_AF_ of situations without periodic AM and without random AM increasing with increasing energy content in the low-frequency range in the order of pink < WT << LF. Within the set of 18 stimuli, the standard deviation of the *L*_AF_ varies from 0.1–2.8 dB, depending on periodic AM, random AM, and spectral shape.

### 2.3. Annoyance Ratings and Questionnaire

Participants were asked to rate their annoyance with the stimuli with the ICBEN 11-point scale [33], with 0 representing the lowest and 10 the highest annoyance rating. To put the annoyance ratings into the context of environmental noise exposure (including WT noise), the participants were asked to answer the same question as in [26] (in German): “When you imagine that this is the sound situation in your garden, what number from 0 to 10 best shows how much you would be bothered, disturbed or annoyed by it?”

The listening tests were complemented with a questionnaire adapted from [26] (Supplementary Materials). The first part contained questions about hearing and well-being, and the second part questions about the participants’ gender, age, living environment, noise sensitivity, attitude towards wind farms, and some concluding questions about the listening test. Noise sensitivity was determined with the NoiSeQ-R [34], which is the Reduced Version of the NoiSeQ [35]. Attitude towards wind farms was measured with the questionnaire of [26].

### 2.4. Laboratory Setup

**Figure 4:**
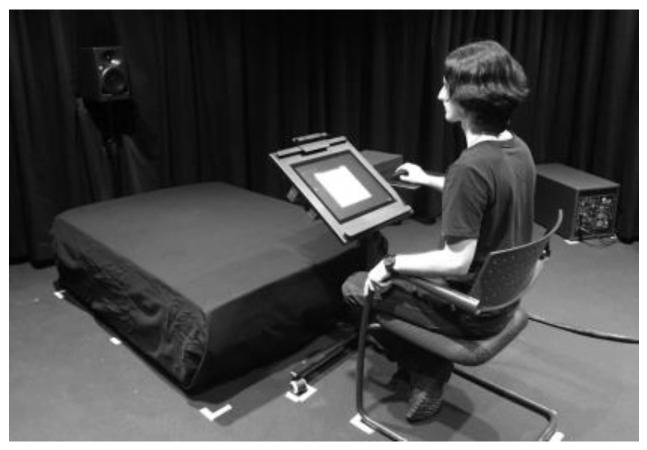
Laboratory setup in AuraLab at Empa, with the listening test program (graphical user interface) displayed on the screen and a porous floor absorber between loudspeaker and participant. One of the two subwoofers is visible on the right side.

The experiments were carried out in the listening test facility AuraLab at Empa (Figure 4). The facility comprises a separate listening and control room, allowing for audio-visual supervision to comply with ethical requirements. The listening room contains a high-quality multichannel loudspeaker reproduction system including a bass management with two subwoofers (Neumann KH 805). It features controlled room acoustics with a reflective floor, low reverberation time (*T*_mid_ = 0.11 s) and low background noise level (7 dBA, GK0).

For the present tests, a one-channel setup together with bass management was chosen. The main loudspeaker (Neumann KH 120 A) was installed at a similar height as and at a distance of 2 m from the seated participant’s head, with a porous floor absorber between the loudspeaker and the participant (Figure 4). The frequency response of the laboratory setup lay within ±3.6 dB for the 1/3 octave bands from 16 Hz to 16 kHz. Prior to the tests, the playback chain was calibrated with a sound level meter located at the position of the seated participant’s head.

### 2.5. Listening Test Procedure

The listening tests were conducted in single sessions as focused tests. The stimuli were played only once, one by one, after complete playback and rating, with a break of 1 s between stimuli. The test procedure consisted of the following steps. First, a short introduction to the research topic and task (annoyance rating of WT and other sounds) was given. Second, the participants signed a consent form to participate. Third, they answered the first part of the questionnaire about hearing and well-being as criteria for study participation. Fourth, they were instructed about the listening test program. Fifth, they did the actual listening test. The test program guided the participants through the test by automatically choosing and playing the stimuli, and by recording the participants’ ratings entered via a graphical user interface (Figure 4). The listening test included (i) an orientation, where the participants listened to five 10 s long stimuli covering the range of situations to be rated, (ii) two exercise ratings, and (iii) the main experiment with annoyance rating of the 20 stimuli, which were reproduced in random order. Finally, the participants completed the second part of the questionnaire.

The whole listening test including the introduction and the questionnaire lasted about one hour. A compensation of 20 Swiss francs (approx. €18) was given for participation.

### 2.6. Participants

Fifty-two participants (24 males, 28 females), aged 18–62 years (median of 43 years), were recruited via online advertisement and word-of-mouth recommendation. The majority worked at Empa. None of the participants wore a hearing aid, and all of them declared that they have normal hearing and feel well (no colds). Sixty-five percent of the participants had heard WT noise before, but none of them lived close to a wind farm.

### 2.7. Statistical Analysis

The consistency of the annoyance ratings across participants was assessed with the inter-rater reliability [36]. To that aim, a two-way random, consistency, average-measures intraclass correlation (ICC) was calculated [37]. Large ICC values indicate a high degree of agreement between participants.

The annoyance ratings were analyzed by means of linear mixed-effects models (e.g., [38]), using the procedure MIXED of IBM SPSS Version 23. In Subset I, *L*_Aeq_ was treated as a continuous variable. In Subset II, depth of periodic AM was treated as a continuous variable, and spectral shape (three situations) and random AM (two situations) as categorical variables. In addition, interactions between the variables of Subset II were studied, as well as the sequence, i.e., the playback number of the stimuli, and the participants’ characteristics (Section 2.3). Further, different random effect models (random intercept; random coefficients describing the dependence on the variables of Table 1) were tested. From the set of potential models, the final model was chosen by considering completeness (include all relevant variables), performance (data representation, significance of effects) and parsimony (keep the model as simple as possible). The models were compared with the Bayesian Information Criterion (BIC) [39], where the model with the lowest BIC is preferred. Compliance with the model assumptions was visually checked with residual plots. The goodness-of-fit of the final model was assessed according to [40,41] with the marginal (*R*^2^_m_) and conditional coefficient of determination (*R*^2^_c_), where *R*^2^_m_ represents the variance explained by the fixed factors and *R*^2^_c_ the variance explained by the fixed plus random factors.

## 3. Results

### 3.1. Descriptive Statistics (Raw Data)

**Figure 5:**
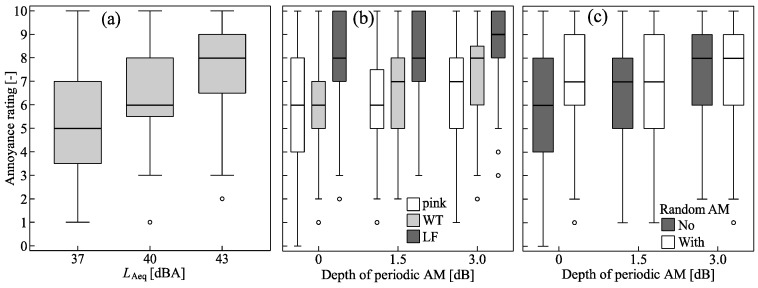
Boxplots of the short-term annoyance ratings as a function of (**a**) the *L*_Aeq_ (Subset I), (**b**) depth of periodic amplitude modulation (AM) and spectral shape (pink, wind turbine (WT), low frequency (LF); pooled data of situations with/without random AM), and (**c**) periodic AM and random AM (pooled data of situations with different spectral shapes) (Subset II). Boxes represent the interquartile range (25% and 75%) and the median (50%, horizontal line in boxes), whiskers the data within 1.5 times the interquartile range, and circles outliers outside the whiskers.

As Figure 5 shows, the individual annoyance ratings of Subsets I and II cover a wide range of the 11-point scale. There is a clear trend of increasing ratings with *L*_Aeq_ (Figure 5a), as well as with spectral shape in the order pink < WT << LF, and with increasing depth of periodic AM (Figure 5b). Further, the annoyance ratings tended to be slightly higher in situations with random AM than in situations without, at least in the absence of periodic AM (Figure 5c). The ICC values for the annoyance ratings of 0.985 (Subset I) and 0.953 (Subset II) lie in the “excellent” range of ICC > 0.75 according to [42] and thus suggest a high degree of agreement between participants [36].

Further, individual ratings of Subset II tended to increase with sequence (playback number) of the stimuli (Pearson correlation coefficient *r* = 0.08, *p* = 0.02). In Subset I, in contrast, no such dependence was found. The ratings were not (strongly) related to the participants’ characteristics gender (*p* = 0.71) or noise sensitivity (*p* = 0.18), but tended to be higher with increasing age (*r* = 0.13, *p* < 0.001) and lower the more positive the attitude towards wind farms (*r* = −0.17, *p* < 0.001).

**Figure 6:**
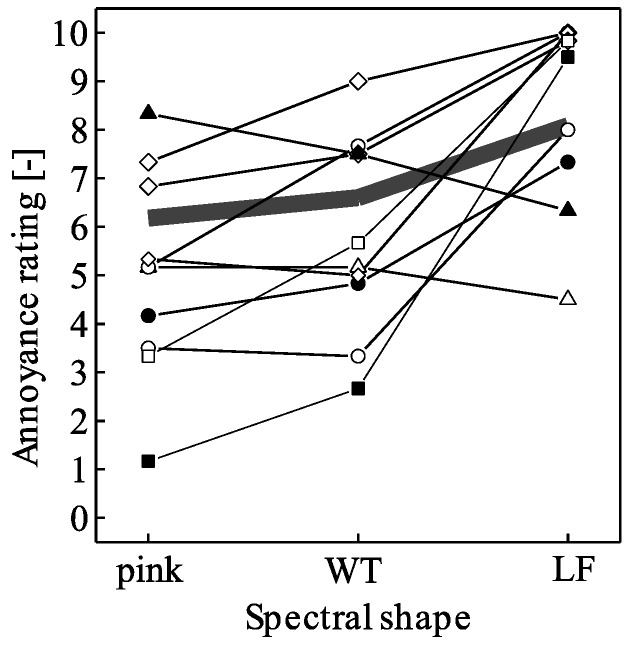
Examples of individual annoyance ratings (mean values per participant and spectral shape, pooled data of situations with/without random and/or periodic amplitude modulation) of 10 randomly selected participants as a function of spectral shape (pink, wind turbine (WT), low frequency (LF)). Different symbols connected by lines represent different participants, and the grey bold line shows the average of all 52 participants.

Since the annoyance ratings are bounded at a value of 10, the difference between the participants’ mean annoyance ratings of LF and pink spectral shapes was negatively correlated to their mean rating of pink shape (*r* = −0.68, *p* < 0.001), i.e., the ratings tended to strongly depend on spectral shape if annoyance to pink shape was low, and vice versa (Figure 6).

### 3.2. Effects of Acoustical Characteristics on Annoyance

**Figure 7:**
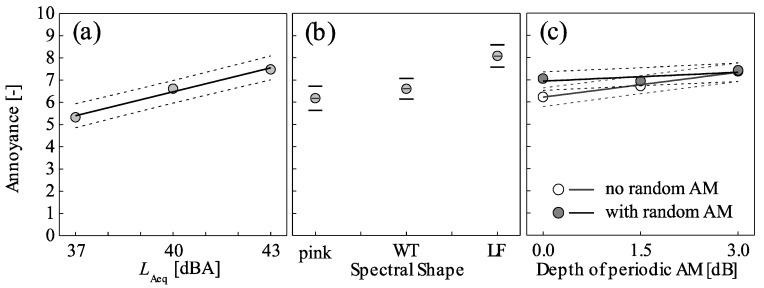
Mean short-term annoyance as a function of (**a**) the *L*_Aeq_ (Subset I), (**b**) spectral shape (pink, wind turbine (WT), low frequency (LF); pooled data of different situations of periodic and random amplitude modulation (AM)), (**c**) and periodic and random AM (pooled data of situations with different spectral shapes) (Subset II). Symbols represent observed values, and lines the corresponding mixed-effects model with 95% confidence intervals, in (**b**) as horizontal lines.

The data of Subset I revealed that annoyance increases linearly with *L*_Aeq_, by more than two units on the 11-point scale for an increase in *L*_Aeq_ from 37 to 43 dBA (Figure 7a). The linear mixed-effects model, which explains more than 80% of the variance (*R*^2^_m_ = 0.19, *R*^2^_c_ = 0.84), confirms the statistical significance of the *L*_Aeq_ (*p* < 0.001) with the following relationship:

*Annoyance* = 0.359 (±0.063) × *L*_Aeq_ − 7.885 (±2.577), (1)

where the numbers in brackets indicate the 95% confidence intervals. Thus, a sound pressure level change of 2.8 dB is associated with a change of 1 unit on the 11-point scale, and vice versa.

The data of Subset II revealed that annoyance strongly increases with increasing energy content in the low-frequency range, in the order pink < WT << LF (Figure 7b). Furthermore, annoyance increases with increasing depth of periodic AM. This effect is very clear in situations without random AM, but less pronounced in situations with random AM (Figure 7c). Finally, annoyance with situations without random AM is lower than with random AM, but only at low depths of periodic AM (Figure 7c).

The observed effects can be described with the following mixed-effects model:

*Annoyance_ijk_* = *μ* + *Spec_i_* + *rAM_j_* + *β* ∙ *pAM_ijk_* + *β_rAM,j_* ∙ *pAM_ijk_* + *γ* ∙ *S_ijk_* + *u_i,k_* + *ε_ijk_*. (2)

In Equation (2), *Annoyance* is the dependent variable, *μ* is the overall mean, *Spec* and *rAM* are the categorical variables spectral shape (3 levels: *i* = 1, 2, 3) and random AM (2 levels: *j* = 1, 2), *pAM* and *S* are the continuous variables periodic AM and sequence, *β* and *γ* are regression coefficients, and *β*_rAM_ represents the interaction between *rAM* and *pAM*. Further, *u_i_*_,*k*_ are the participants’ random coefficient terms (*k* = 1, …, 52). They account for the dependence of the individual annoyance ratings on spectral shape (Figure 6), using an unstructured covariance matrix for that purpose. Finally, the error term *ε* is the random deviation between observed and predicted values of *Annoyance*. The index *ijk* represents the *k*th replicate observation of the *i*th spectral shape at the *j*th random AM.

**Figure 8:**
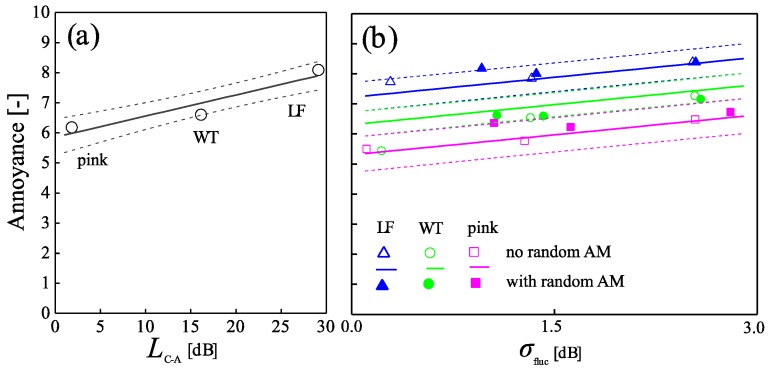
Mean short-term annoyance of Subset II as a function of (**a**) the sound level difference *L*_C-A_ and (**b**) standard deviation of the A-weighted and FAST time-weighted high-pass filtered (*f* > 500 Hz) level-time histories of the signals (*σ*_fluc_). Symbols represent observed values, in (**b**) as mean values per stimulus, and lines the corresponding mixed-effects model with 95% confidence intervals.

In addition to the variables of Equation (2), the interaction between *Spec* and *pAM* was significant (*p* < 0.001), but seemed to be primarily caused by the outlying (low) rating to the stationary WT stimulus (lowest rating to WT in Figure 8b). Further, also the participants’ attitude towards wind farms was significant (*p* = 0.02). It was, however, not of focus here. The other tested variables (interaction terms *Spec* × *rAM* × *pAM* and *Spec* × *rAM*; participants’ gender, age and noise sensitivity) were not significant (*p* = 0.09–0.90). None of these variables were included in the model. The final model explains a large part of the variance (*R*^2^_m_ = 0.18, *R*^2^_c_ = 0.81), although only ~20% with the fixed effects. The model parameters are given in Table A1 of the Appendix.

Figure 7 further reveals the following variation in annoyance for the acoustical characteristics and equivalents of a (WT) sound pressure level change. On average, a change in spectral shape from pink to LF increases annoyance by almost two units on the 11-point scale (Figure 7b). The same effect would be evoked by a level increase of 5.3 dB. The effects of periodic and random AM are less pronounced. On average, an increase in depth of periodic AM from *σ*_pAM_ = 0 to 3 dB increases annoyance by more than 1 unit in situations without random AM, but only by 0.4 units with random AM. This would also be evoked by a level increase of 3.1 and 1.1 dB, respectively (Figure 7c). Similarly, in the absence of periodic AM, annoyance with situations with random AM on average is 0.7 units higher than without random AM. This corresponds to a level increase of 2.0 dB. No such effect, in contrast, is observable in situations with strong periodic AM. Finally, the mean annoyance with the individual stimuli of Subset II covers a wide range of three units on the 11-point scale (cf. Figure 8b). This corresponds to an equivalent level change of 8.4 dB.

### 3.3. Explorative Data Re-Analysis

While the acoustical variables of Table 1 affect short-term annoyance, they are usually not easy to determine, especially in the case of field recordings. Therefore, the stimuli of Subset II were characterized with two more accessible variables as substitutes for those of Table 1, to study their effects on annoyance:

- ***L*_C-A_** ≡ sound level difference *L*_Ceq_–*L*_Aeq_ (cf. Section 2.2): indicator for the low-frequency content of the stimuli and substitute for the variable spectral shape.
- ***σ*_fluc_** ≡ standard deviation of the A-weighted, FAST time-weighted level-time histories of the high-pass filtered stimuli: indicator for the level variation due to periodic and random AM and substitute for the two variables. A high-pass filtered signal (here, with a cutoff frequency of 500 Hz, i.e., *f* > 500 Hz) was used to minimize the influence of spectral shape on level variation (Figure 3) and to obtain approximate independence of the two variables *L*_C-A_ and *σ*_fluc_.

Using these variables, Subset II was re-analyzed. Figure 8 shows the results. Both *L*_C-A_ and *σ*_fluc_ are strongly associated with short-term annoyance.

The observed effects can be described with the following mixed-effects model:

*Annoyance_k_* = *μ* + *δ* ∙ *L*_C-A_,*_k_* + *η* ∙ *σ*_fluc,*k*_ + *γ* ∙ *S_k_* + *u*_0*k*_ + *u*_1*k*_ + *ε_k_*, (3)

where *Annoyance*, *μ*, *γ*, *S*, *ε* and the index *k* are defined in Equation (2), *L*_C-A_ and *σ*_fluc_ are the continuous variables introduced above, *δ* and *η* are regression coefficients, and the terms *u*_0*k*_ and *u*_1*k*_ are the participants’ correlated random intercept and slope (unstructured covariance matrix) to account for the dependence of the individual ratings on *L*_C-A_, in analogy to spectral shape in Equation (2). The variables included in the model are all highly significant (*L*_C-A_ and *σ*_fluc_: *p* < 0.001; *S*: *p* < 0.002). The model parameters are given in Table A2 of the Appendix. An analogous model can also be established for the spectral slope variable (cf. Section 2.2) instead of *L*_C-A_.

Although the model of Equation (3) is considerably simpler than the one established for spectral shape, periodic and random AM (Equation (2)), using only four instead of seven degrees of freedom for the fixed effects, it represents the data equally accurately, with very similar coefficients of determination (*R*^2^_m_ of 0.16, *R*^2^_c_ of 0.74). Further, the model has the advantage that all variables are continuous and thus allow for interpolation and (to some degree) extrapolation of the results.

## 4. Discussion

In this study, a laboratory listening experiment was performed using stimuli representing different situations of WT and other, generic broadband sounds. The factorial design in combination with the sound synthesis tools used to generate the stimuli and the statistical methods allowed for separation of the relative contributions of the three acoustical characteristics spectral shape, depth of periodic AM and random AM to short-term annoyance. Further, with the study design the variation in annoyance reactions to the acoustical situations could be expressed as equivalent changes in (WT) sound pressure level, which is the most often used indicator in noise exposure assessments.

### 4.1. Acoustical Characteristics and Annoyance

Annoyance was found to increase with sound pressure level (here, *L*_Aeq_). This was expected and reported in many previous laboratory (e.g., [15,26,27]) as well as field studies (e.g., [5,6]). Besides the *L*_Aeq_, also the acoustical characteristics of Table 1 were found to be strongly linked to annoyance. Similar annoyance changes would be evoked by equivalent sound pressure level changes of more than 8 dB, corresponding to more than a 6-fold change in sound energy. Thus, different amplitude modulated broadband noises at a constant *L*_Aeq_ of 40 dBA were perceived very differently with respect to annoyance, even without presence of tonal or impulsive components, which are well known to be strongly linked to annoyance (e.g., [43,44]).

First, spectral shape was found to be important. Annoyance increased with increasing energy in the low-frequency range (Figure 7). This effect was also found in other studies [8,9]. It could be well predicted with the variable *L*_C-A_ (Figure 8), which was also found in [11]. The latter study assessed annoyance as “pink noise equivalents” and found an increase of 0.46 dB per 1 dB *L*_C-A_, while our study yields “WT noise equivalents” with an increase of 0.20 dB per 1 dB *L*_C-A_. Interestingly, while WT was found here to be more annoying than pink spectral shape (~1.2 dB equivalent sound pressure level change: Figure 7), another laboratory study [27] found WT to be ~5 dB more annoying than pink noise if participants were residents of wind farms, but vice versa if they were not. Further, contrasting our results, other studies stress the relative importance of high compared to low frequencies [12,13]. Second, periodic AM was associated with annoyance. Also this observation is in line with the literature [15,16,26]. Third, as with periodic AM, annoyance increased with random AM. So far, this effect was not systematically studied in literature. Nevertheless, it is consonant with the results of [26] insofar as the latter revealed that annoyance with AM is not related to its periodicity but rather to the modulation frequency range. Here, the effects of periodic and random AM were not only of similar magnitude but also interrelated, i.e., annoyance increased less with increasing periodic AM in situations with random AM than without, and vice versa (Figure 7). Further corroborating the similarity of effects, periodic and random AM could be combined into a single variable for level fluctuations to predict annoyance without a notable loss of accuracy (Figure 8).

### 4.2. Sensory Perception and Annoyance

The participants’ annoyance ratings were found to be closely linked to their sensory perception, as answers to the concluding questions of the questionnaire (Supplementary Materials) revealed: Several participants mentioned discomfort due to a “*pulsing*” sensation in the ears by the “*dull*” or “*low*” LF sounds. The “*pulsing*” sensation may also have been caused by periodic AM. Besides, some participants found “*hissing*” or “*high*” sounds annoying, which might have referred to pink spectral shape, but probably also to the short-time level fluctuations of random AM. Thus, the sensory perception seems to be an important link between the acoustical characteristics of the stimuli and annoyance, which may help to better understand and predict annoyance reactions to noise. These aspects can be further explored with semantic differential tests (e.g., [45]). For that purpose, a corresponding test was carried out and is being analyzed with the stimuli of Subset II (work in progress).

Similarly, instead of sound pressure level-related quantities, psychoacoustic parameters [28] might serve as alternative, more precise predictors for aural perception and thus annoyance. Here, loudness seems particularly important. Short-term annoyance was reported to be strongly correlated with perceived or calculated loudness (e.g., [46,47,48]). Further, a listening experiment on road traffic noise revealed loudness to be a good predictor for annoyance with stimuli varying in *L*_Aeq_ and *L*_C-A_ [11]. Nevertheless, short-term annoyance and loudness are not the same. In fact, in the present listening experiment, annoyance was found to increase with calculated Zwicker loudness [49] for the stimuli of Subset I where only the *L*_Aeq_ was varied, but to decrease with Zwicker loudness for the stimuli of Subset II comprising different acoustical characteristics (details see [19]). Apparently, Zwicker loudness was inappropriate to predict annoyance with the studied sounds. Analogous results were found in another laboratory study with low loudness levels [50]. Other loudness models may be more appropriate. Besides, further psychoacoustic parameters may be important, such as fluctuation strength in the case of the present data set. Fluctuation strength describes the hearing sensation of AM at fluctuation frequencies below 20 Hz [28]. It has been suggested that it reaches its maximum at 4 Hz [51]. Fluctuation strength might describe the (combined) perception of periodic and random AM and thus be an appropriate predictor for their associated annoyance. Re-analysis of the data sets with calculated psychoacoustic parameters might thus yield additional insights.

These are possible future research approaches to establish a closer link between acoustical characteristics, perception, and short-term annoyance.

### 4.3 Annoyance Responses in Laboratory vs. Field Studies

In interpreting the present findings, one needs to consider the inherent differences between short-term annoyance investigated in the laboratory in a focused test and annoyance with long-term exposure in the field [48]. In particular, annoyance in the laboratory is usually closely associated with the *L*_Aeq_ (Section 4.1) and also with loudness parameters (Section 4.2). It thus seems to be closely related to the sensory perception of sound. Supporting this interpretation, the participants’ ratings in the present study were highly consistent (ICC values > 0.9).

In field studies (socio-acoustic surveys), the association of annoyance with *L*_Aeq_ (or related noise metrics) is usually weaker than in laboratory studies, with values of *R*^2^ being in the range of 0.05–0.25 [52]. This might be related to various reasons. First, noise calculations usually reflect outdoor exposure (e.g., [5,6]), while study participants spend much of their time indoors. However, low frequencies are attenuated less than high frequencies during sound transmission (e.g., [29]), which changes spectral shape and increases *L*_C-A_. Second, noise calculations are afflicted with uncertainties ranging from ~1 dB (aircraft noise [53]) to ~4 dB (WT noise at distances <1 km [54]). Third, temporal patterns such as short-time level variations (AM) are usually neglected in calculations. Fourth, people commute between home, work and leisure and are thus exposed to a variety of sound situations (and not to specific situations as in the laboratory), which is never accounted for. Field studies thus carry a large exposure misclassification bias, which reduces the strength of statistically modelled relationships. Fifth, in the field not only the noise source in focus for annoyance, but also other sources are present as effect modifiers. This may affect annoyance, be it by an improved acoustic quality [55] or by masking [56,57]. Finally, personal and situational factors may strongly influence annoyance in the field [5,58], but probably less so in the laboratory (as, e.g., in this study or in [26]). These are all aspects that explain the higher correlations one sees in the laboratory.

In conclusion, while the present study design allowed disclosing the relative contributions of different acoustical characteristics to annoyance without potential effect modifiers, the high experimental control (including generic stimuli) was at the expense of ecological validity. Field studies, in contrast, have high ecological validity, but at the expense of control (sound exposure, effect modifiers). Thus, laboratory studies are best for reliable effect differentiations, which then might be validated in field studies. Consequently, laboratory studies should precede field studies—and not vice versa.

### 4.4. Practical Implications for WT Noise

Keeping the above discussed limited ecological validity of the present results in mind, practical implications may be tentatively directed towards WT noise. Given the important role of acoustical characteristics of (WT) broadband noise for annoyance, the design, operation and noise assessment of WTs and wind farms may positively or negatively affect residents in terms of annoyance. First, acoustically optimized designs of WT rotor blades may reduce sound emission and thus exposure [17]. Second, larger WTs may emit more low-frequent sound [29]. However, changes in spectral shape are much less pronounced than in the situations studied here, and a recent study suggested that the size of WTs might not be relevant for annoyance [59]. Third, annoyance might be reduced by reducing the occurrence of periodic AM by blade pitch control [60] and by prevention of stall on the WT blades [14]. On the other hand, periodic AM, if occurring, may be enhanced by interference between WTs of a wind farm [61]. Fourth, operational restrictions such as limits of angular blade velocity as a function of the wind direction may effectively reduce annoyance while still allowing for cost-effective energy production [16]. Fifth, the important role of the acoustical characteristics of WT noise asks for reliable assessed methods. A recent procedure allows measuring WT noise even in presence of masking sounds [62]. Finally, policy makers should account (more) for specific acoustical characteristics of WTs, e.g., by adding a penalty for periodic AM [63]. Current legislation distinctly varies between countries [64,65].

## 5. Conclusions

In the present laboratory listening experiment, the effects of three acoustical characteristics of broadband noise on short-term annoyance were studied, namely, spectral shape, depth of periodic AM, and random AM. To that aim, realistic WT as well as generic broadband sounds with pink and LW spectral shape were presented in a listening experiment, and participants’ annoyance reactions were recorded. The full factorial design of the listening experiment allowed for separation of the relative contributions of the acoustical characteristics to annoyance. It was found that besides sound pressure level, all three studied characteristics affect annoyance: Annoyance increased with increasing energy content in the low-frequency range as well as with depth of periodic AM, and was higher in situations with random AM than without. Similar annoyance changes would be evoked by sound pressure level changes of up to 8 dB. Thus, in essence, we could demonstrate that besides standard sound pressure level metrics spectral shape as well as short-term temporal level variations (i.e., AM) should be considered in environmental impact assessments. Our findings are particularly important in the wake of environmental impact assessments for WT noise. They cater to manufacturers and policy makers alike—people who want to forecast residents’ annoyance near such installations.

## Figures and Tables

**Table 1:** Factorial design of the listening experiment with acoustical stimuli of different spectral shapes (low-frequency (LF), wind turbine (WT), pink), depth of periodic amplitude modulation (AM, expressed as standard deviation (*σ*_pAM_) of the periodic level fluctuations), and occurrence or absence of random AM (with, no). The table shows the number of stimuli per variable combination. Except for two stimuli (cf. footnote 1), the stimuli were reproduced at a *L*_Aeq_ of 40 dBA.

Depth of Periodic AM				Spectral Shape				
	LF			WT			Pink	
				Random AM				
	with	no		with	no		with	no
*σ*_pAM_ = 0.0 dB	1	1		3 ^1^	1		1	1 ^2^
*σ*_pAM_ = 1.5 dB	1	1		1	1		1	1
*σ*_pAM_ = 3.0 dB	1	1		1	1		1	1

^1^ reference stimulus, additionally reproduced at a *L*_Aeq_ of 37 and 43 dBA, besides 40 dBA. ^2^ pink noise.

## Supplementary Materials

The following are available online at http://www.mdpi.com/1660-4601/15/5/1029/s1, Supplementary File: Authors’ questionnaire (in German). Note that page 6 of the questionnaire partly refers to the listening test mentioned in Section 4.2, which was carried out along with the experiment described here.

## Appendix A

**Table A1:** Model coefficients with 95% confidence intervals (CI) and probabilities (*p*) of the linear mixed-effects model for short-term annoyance (Equation (2)).

		Linear Mixed-Effects Model		
Variable ^1^	Symbol ^1^	Coefficient	95% CI	*p*
Intercept	*µ*	6.4028	[5.8998; 6.9057]	<0.001
Spectral shape	*S* *pec_i_*_=LF_	1.4736	[0.9491; 1.9981]	<0.001
	*S* *pec_i_*_=WT_	0 ^2^		
	*S* *pec_i_*_=pink_	−0.4307	[−0.7848; −0.0766]	0.02
Random AM	*rAM_j_*_=no_	−0.7238	[−0.9345; −0.5131]	<0.001
	*rAM_j_*_=with_	0 ^2^		
Periodic AM	*β*	0.1330	[0.0563; 0.2098]	<0.001
Random × Periodic AM	*β*_rAM,*j*=no_	0.2424	[0.1335; 0.3513]	<0.001
	*β*_rAM,*j*=with_	0 ^2^		
Sequence	*γ*	0.0178	[0.0055; 0.0301]	<0.005
Random effect variance(spectral shape)	*u* ^2^ *_i_*_=LF,*k*_	3.1156	[2.0670; 4.6961]	<0.001
	*u* ^2^ *_i_*_=WT,*k*_	2.6436	[1.7469; 4.0006]	<0.001
	*u* ^2^ *_i_*_=pink,*k*_	3.6389	[2.4219; 5.4674]	<0.001
Residual variance	*ε* ^2^ *_ijk_*	1.0664	[0.9654; 1.1780]	<0.001

^1^ Variables and symbols: see Equation (2). ^2^ Redundant coefficients are set to zero.

**Table A2:** Model coefficients with 95% confidence intervals (CI) and probabilities (*p*) of the linear mixed-effects model for short-term annoyance (explorative data re-analysis, Equation (3)).

		Linear Mixed-Effects Model		
Variable ^1^	Symbol ^1^	Coefficient	95% CI	*p*
Intercept	*µ*	4.9498	[4.3186; 5.5811]	<0.001
*L*_C-A_	*δ*	0.0702	[0.0455; 0.0949]	<0.001
*σ*_fluc_	*η*	0.4436	[0.3553; 0.5319]	<0.001
Sequence	*γ*	0.0215	[0.0081; 0.0349]	<0.002
Random effect variance (intercept and slope)	*u* ^2^_0*k*_	4.4574	[2.9644; 6.7023]	<0.001
	*u* ^2^_1*k*_	0.0073	[0.0048; 0.0111]	<0.001
Residual variance	*ε* ^2^ *_ijk_*	1.3669	[1.2415; 1.5050]	<0.001

^1^ Variables and symbols: see Equation (3).
