# Supplementary material for: Effects of Different Spectral Shapes and Amplitude Modulation of Broadband Noise on Annoyance Reactions in a Controlled Listening Experiment
Source: Int J Environ Res Public Health. 2018 May 19;15(5):1029. doi: 10.3390/ijerph15051029 (PMC5982068; doi:10.3390/ijerph15051029)
Supplement: Supplementary file 1 [file ijerph-15-01029-s001.pdf]

Empa
Materials Science and Technology

PersID:

Studie zur Wahrnehmung und Lästigkeit von Windturbinengeräuschen

Fragebogen

Hörvermögen und Wohlbefinden	PersID:

Anleitung zum Ausfüllen

Im Folgenden möchten wir Sie bitten, Fragen zu Hörvermögen und Wohlbefinden zu beantworten. Kreuzen Sie die Antwort an, die am besten auf Sie zutrifft. Wenn Sie sich nicht sicher sind, welche Antwortmöglichkeit Sie ankreuzen sollen, wählen Sie bitte diejenige, die am ehesten auf Sie zutrifft. Sie dürfen für jede Aussage nur **ein** Kreuzchen vergeben.

Hörvermögen

1. Wie gut ist Ihrer Schätzung nach Ihr Hörvermögen?

☐ Sehr gut ☐ Gut ☐ Mittelmässig ☐ Schlecht ☐ Sehr schlecht

2. Litten oder leiden Sie an einer Ohrerkrankung?

☐ Ja ☐ Nein

3. Hatten Sie einen Schädelunfall mit Folgen für das Gehör?

☐ Ja ☐ Nein

4. Erlitten Sie einen Schiess- oder Explosionsschaden (Knalltrauma)?

☐ Ja ☐ Nein

5. Benutzen Sie einen Hörapparat?

☐ Ja ☐ Nein

6. Müssen/mussten Sie bei der Arbeit einen Gehörschutz tragen?

☐ Ja ☐ Nein

7. Leiden Sie unter Ohrgeräuschen (Ohrensausen, Tinnitus)?

☐ Ja ☐ Nein

8. Sind Sie zurzeit erkältet?

☐ Ja ☐ Nein

Wohlbefinden

9. Fühlen Sie sich zurzeit gesund und wohl?

☐ Ja ☐ Nein, nämlich: . . . .

10. Fühlen Sie sich zurzeit sehr müde?

☐ Ja ☐ Nein

Aussagen zu Geräuschen

PersID:

Anleitung zum Ausfüllen

Im Folgenden möchten wir Sie bitten, Aussagen zu verschiedenen Geräuschen zu machen. Gehen Sie bitte die Aussagen der Reihe nach durch und lassen Sie keine aus.
Versuchen Sie bitte, sich in die jeweilige Situation hineinzuversetzen, und antworten Sie spontan, ohne lange zu überlegen, ob Sie der Aussage **im Allgemeinen** zustimmen oder nicht.
Kreuzen Sie die Antwortmöglichkeit an, die **Ihrer Meinung nach** am besten auf Sie zutrifft. Sie dürfen für jede Aussage nur **ein** Kreuzchen vergeben.
Uns interessiert Ihre ganz persönliche Meinung zu den Aussagen, die wir Ihnen vorgeben. Aus diesem Grunde gibt es auch keine richtigen oder falschen Antworten. Wenn Sie sich nicht sicher sind, welche Antwortmöglichkeit Sie ankreuzen sollen, wählen Sie bitte diejenige, die am ehesten auf Sie zutrifft.

		**Stimmt genau**	**Stimmt eher**	**Stimmt eher nicht**	**Stimmt gar nicht**
**1.**	**Gesunder Schlaf ist für mich nur in absolut ruhiger Umgebung möglich.**				
**2.**	**Neue Aufgaben kann ich nur in leiser Umgebung bearbeiten.**				
**3.**	**Ich kann mich schnell an Lärm in meiner Wohnumgebung gewöhnen.**				
**4.**	**Ich werde sehr unruhig, wenn ich beim Einschlafen jemanden reden höre.**				
**5.**	**Ich bin sehr empfindlich gegenüber Geräuschen aus meiner Nachbarschaft.**				
**6.**	**Wenn Personen um mich herum laut sind, komme ich mit meiner Arbeit nicht voran.**				
**7.**	**Ich bin geräuschempfindlich.**				
**8.**	**Meine Leistung wird durch eine große Geräuschkulisse stark beeinträchtigt.**				
**9.**	**Wenn es nachts laut ist, bin ich morgens unausgeschlafen.**				
**10.**	**Es würde mir nichts ausmachen, an einer lauten Straße zu wohnen.**				
**11.**	**Für eine ruhige Wohnlage nehme ich andere Nachteile in Kauf.**				
**12.**	**Für anstrengende Arbeiten brauche ich äußerste Ruhe.**				
**13.**	**Ich kann einschlafen, obwohl es laut um mich herum ist.**				

**Fragen zur Einstellung**	PersID:

Anleitung zum Ausfüllen

Im Folgenden möchten wir Sie bitten, Aussagen zu Ihrer Einstellung zu Windkraftanlagen zu machen. Gehen Sie bitte die Aussagen der Reihe nach durch und lassen Sie keine aus.
Antworten Sie bitte spontan, ohne lange zu überlegen, ob Sie der Aussage **im Allgemeinen** zustimmen oder nicht.
Kreuzen Sie die Antwortmöglichkeit an, die **Ihrer Meinung nach** am besten auf Sie zutrifft. Sie dürfen für jede Aussage nur **ein** Kreuzchen vergeben.
Uns interessiert Ihre ganz persönliche Meinung zu den Aussagen, die wir ihnen vorgeben. Aus diesem Grunde gibt es auch keine richtigen oder falschen Antworten. Wenn Sie sich nicht sicher sind, welche Antwortmöglichkeit Sie ankreuzen sollen, wählen Sie bitte diejenige, die am ehesten auf Sie zutrifft.

		**Stimmt genau**	**Stimmt eher**	**Weder/ noch**	**Stimmt eher nicht**	**Stimmt gar nicht**
**1.**	**Ich finde Windkraftanlagen gut.**					
**2.**	**Windkraftanlagen sind nützlich für die Gesellschaft.**					
**3.**	**Ich würde nicht in die Nähe von Windkraftanlagen ziehen.**					
**4.**	**Windkraftanlagen sind ungesund für Anwohner.**					
**5.**	**Ich würde für den Ausbau von Windkraftanlagen stimmen.**					
**6.**	**Windkraftanlagen tragen zur Umweltverschmutzung bei.**					
**7.**	**Windkraftanlagen wirken auf mich bedrohlich.**					
**8.**	**Windkraftanlagen stören die Landschaft.**					
**9.**	**Ich wäre bereit, für die Förderung von Windkraftanlagen mehr zu bezahlen.**					
**10.**	**Windkraftanlagen nerven mich.**					

Fragen zu Ihrer Person

PersID:

Worauf sollte die Schweizer Politik in Zukunft stärker achten (bitte genau **1 Kreuzchen** vergeben)?

☐ auf den Wirtschaftsstandort Schweiz

oder

☐ auf Ruhe und Umweltschutz

Allgemeine Angaben:

Geschlecht: ☐ weiblich
☐ männlich

Alter: ☐☐

Wohnlage (bitte **pro Zeile** genau **1 Kreuzchen** vergeben):

**1.**	☐ eher städtisch	☐ eher ländlich
**2.**	☐ eher laut	☐ eher leise
**3.**	☐ in der Nähe einer Windkraftanlage	☐ fernab von Windkraftanlagen

Ich besitze einen Gartensitzplatz oder einen Balkon/Terrasse: ☐ nein
☐ ja, nämlich: . . . .

Ich habe Windturbinenlärm bereits einmal gehört: ☐ nein
☐ ja

Ich war schon einmal in einem Windpark: ☐ nein
☐ ja

Abschliessende Bemerkungen

PersID:

Zum Schluss können Sie in Ihren eigenen Worten allgemeine Bemerkungen zum Versuch machen. Auf der Rückseite hat es weiteren Platz für Bemerkungen.

Fanden Sie irgendetwas an den Geräuschen im Hörversuch besonders lästig, z.B. eine bestimmte Geräuschcharakteristik?

☐ nein

☐ ja, nämlich: . . . .

Hat sich während des Versuches Ihre Art zu Urteilen geändert?

Hörversuch Teil 1: ☐ nein

☐ ja, nämlich: . . . .

Falls ja: Hatte dies einen Einfluss auf Ihre Urteile in Hörversuch Teil 2?

☐ nein

☐ ja, nämlich: . . . .

Hörversuch Teil 2: ☐ nein

☐ ja, nämlich: . . . .

Was können Sie generell zum Versuch berichten? Wie empfanden Sie ihn? War der Versuch z.B. interessant, zu lange, wie war die Atmosphäre im Labor, war der Versuch «in Ordnung», …?

. . . .

. . . .

. . . .

Haben Sie zusätzliche Gedanken, Ideen oder Anregungen, die Sie anfügen möchten?

. . . .

. . . .

Damit ist der Versuch nun beendet. Vielen Dank für Ihre Teilnahme!
